# Supplementary material for: Lineage structure of Streptococcus pneumoniae may be driven by immune selection on the groEL heat-shock protein
Source: Sci Rep. 2017 Aug 22;7:9023. doi: 10.1038/s41598-017-08990-z (PMC5567354; doi:10.1038/s41598-017-08990-z)
Supplement: Supplementary file 1 — Supplementary Figures and Gene Description [file 41598_2017_8990_MOESM1_ESM.pdf]

Lineage structure of *Streptococcus pneumoniae* may be driven by immune selection on the groEL heat-shock protein.

José Lourenço*[,a], Eleanor R. Watkins[a], Uri Obolski[a], Samuel J. Peacock[a], Callum Morris[b], Martin C. J. Maiden[a], Sunetra Gupta[a]

a Department of Zoology, University of Oxford, Oxford, OX1 3PS, United Kingdom
b University of Durham, Durham, United Kingdom

*** jose.lourenco@zoo.ox.ac.uk**

Supplementary Figures

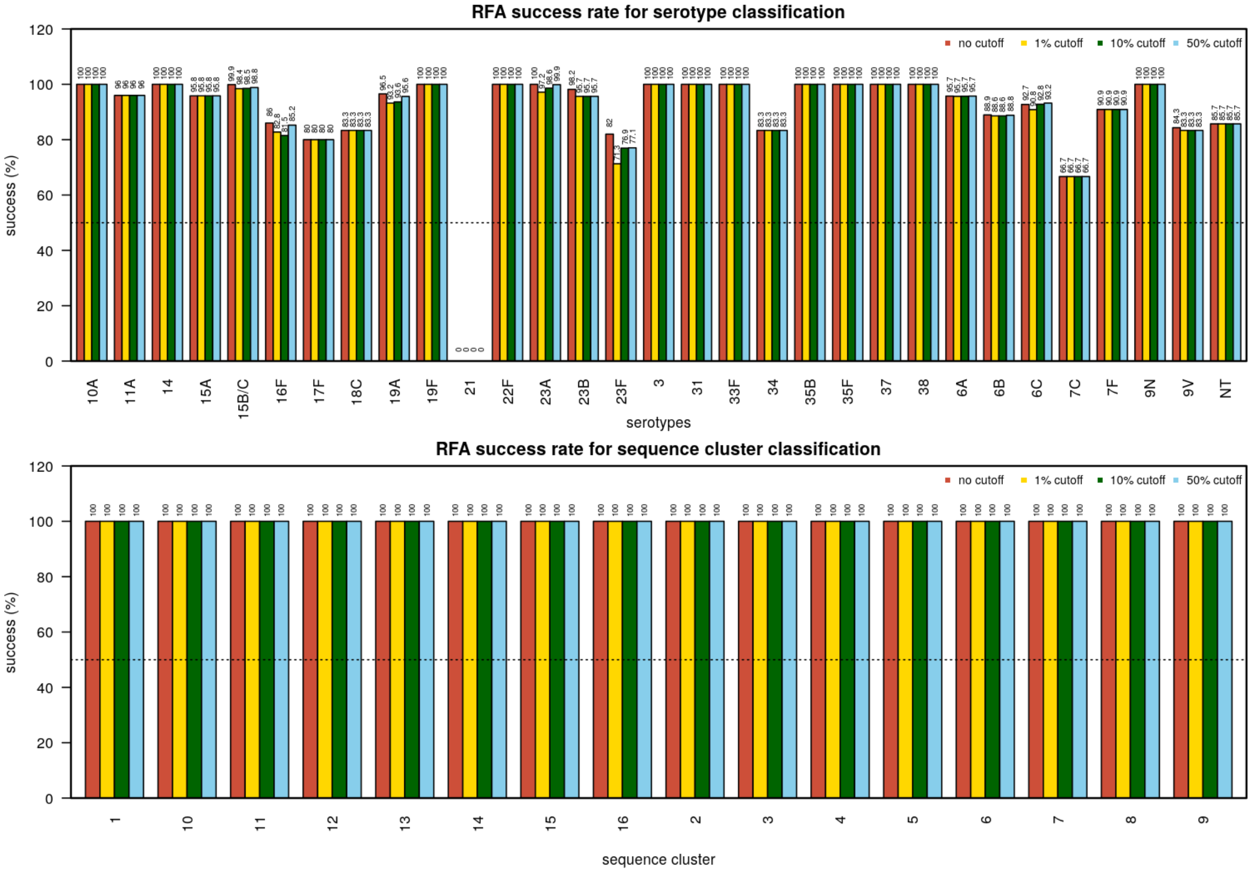

Figure 1: **Random forest classification success rates across experiments presented in the main text.** Results from experiments are presented in different colors, in blue when using 50% cutoff of gene mismatches, green using 10%, yellow 1% and red when no cutoff is used. *(top)* Success rates for serotype classification. *(bottom)* Success rates for Sequence Cluster (SC) classification.

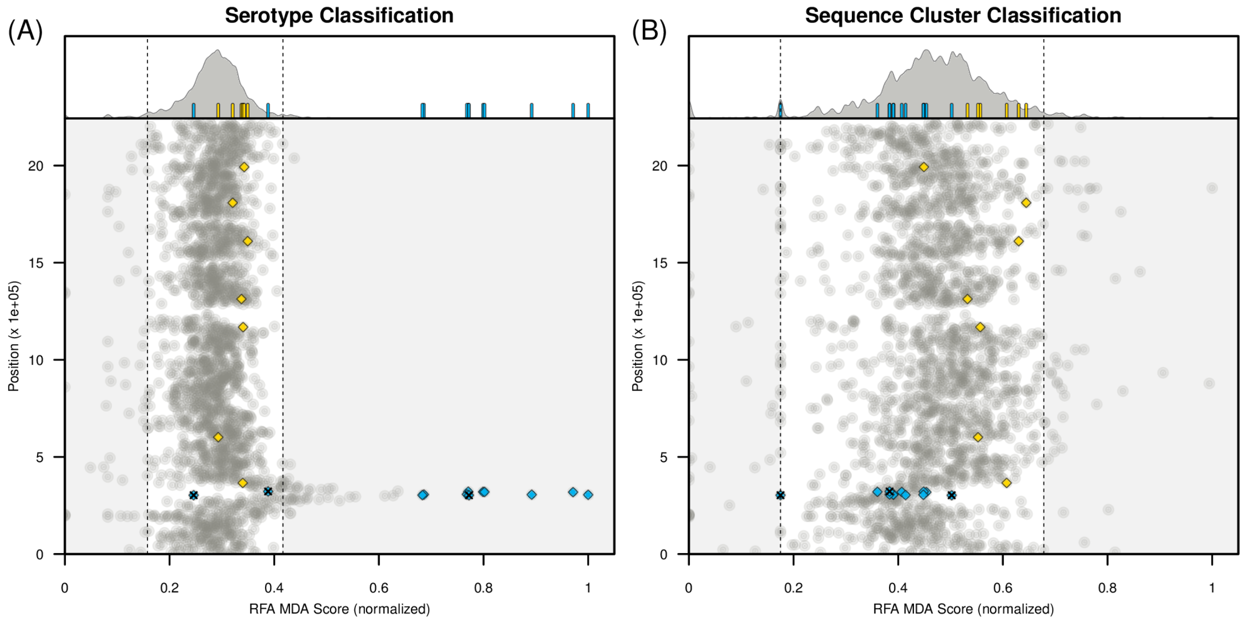

Figure 2: **Serotype and sequence cluster classification for 50% cutoff of gene mismatches.** *(A)* Random forest analysis for serotype classification. *(top)* Density function of normalised RFA scores (normalized to 1 using the maximum score). The 95% boundaries of the distribution are marked by the dashed lines. *(bottom)* Genomic position for each gene in the dataset against their normalised RFA score. The circular genome is presented in a linear form, with the first gene at position 186 being dnaA and the last gene at position 2220530 being parB. MLST genes are marked in yellow circles (*spi, xpt, glkA, aroE, ddlA, tkt*). Genes within the capsular locus are marked with blue squares, with pseudo genes further marked with an 'x'. *(B)* Random forest analysis for sequence cluster classification. Figure details the same as in *A*.

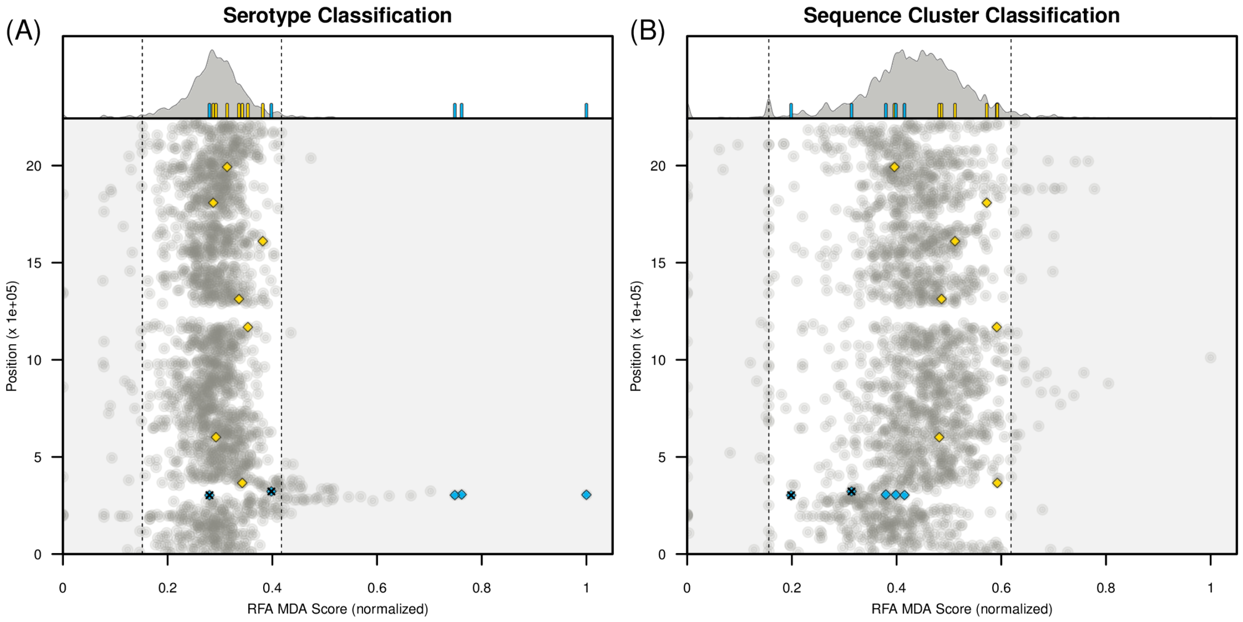

Figure 3: **Serotype and sequence cluster classification for 10% cutoff of gene mismatches.** *(A)* Random forest analysis for serotype classification. *(top)* Density function of normalised RFA scores (normalized to 1 using the maximum score). The 95% boundaries of the distribution are marked by the dashed lines. (*bottom)* Genomic position for each gene in the dataset against their normalised RFA score. The circular genome is presented in a linear form, with the first gene at position 186 being dnaA and the last gene at position 2220530 being parB. MLST genes are marked in yellow circles (*spi, xpt, glkA, aroE, ddlA, tkt*). Genes within the capsular locus are marked with blue squares, with pseudo genes further marked with an 'x'. *(B)* Random forest analysis for sequence cluster classification. Figure details the same as in *A*.

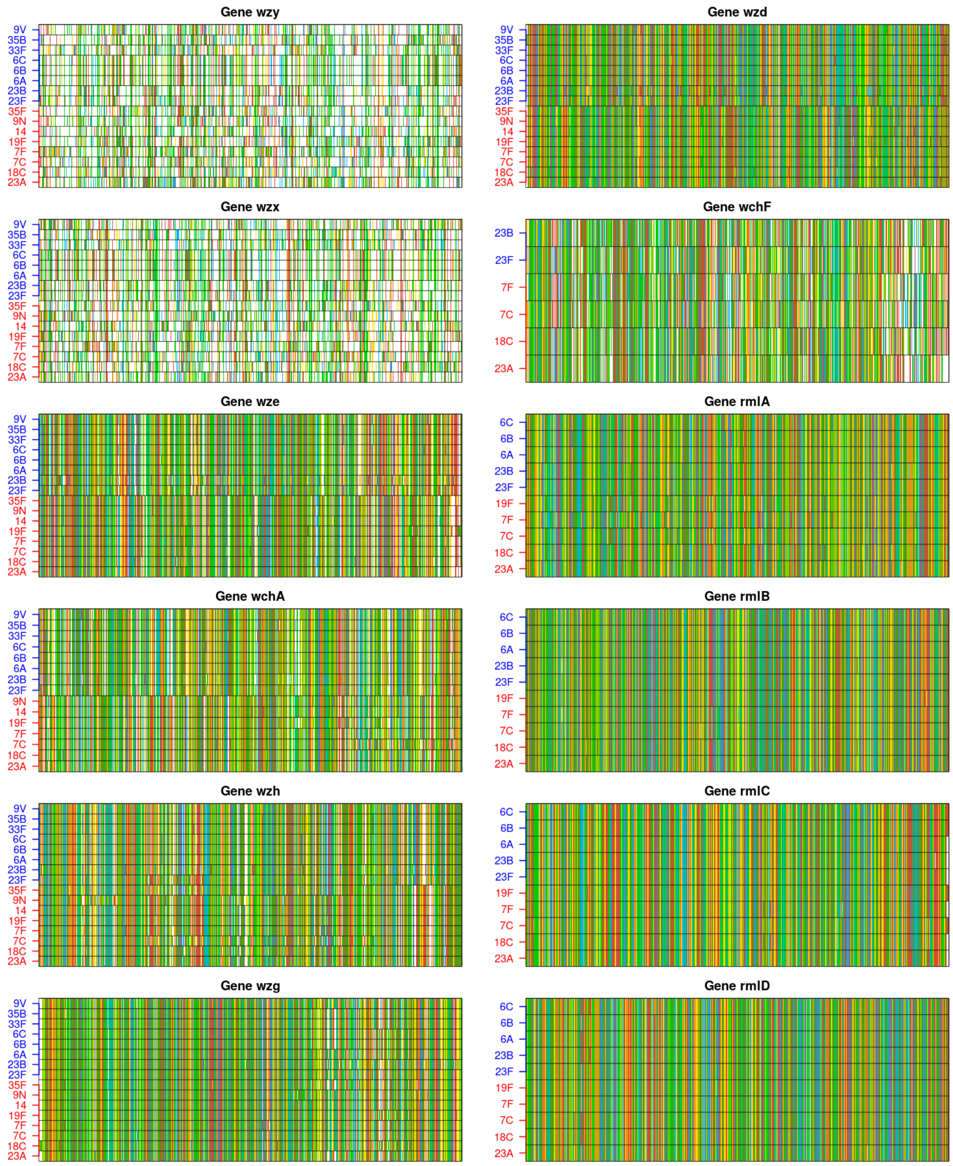

Figure 4: **Alignment of genes within the capsular locus.** The consensus sequence for each capsular gene within a serotype was found by determining the most frequent base per position. Genes were aligned across serotype using a highly restrictive routine penalizing the introduction of gaps (MUSCLE software parameters: -gapopen -12.0 -gapextend -1.0). Gaps are shown in white, Adenine in red, Thymine in green, Cytosine in blue, and Guanine in yellow. Each gene is only represented by the serotypes for which sequences were found; and genes for which 3 or less serotypes were represented are not displayed ($wchV$, $wchW$, $wchX$). The red/blue colours herein used in serotype labels mirror Varvio et al. serotype categories according to statistically, highly supported sequence clusters found in that study. Accordingly, we find genes *wzg* and *rmlABCD* to present no population structure, while genes *wzh*, *wzd*, *wze* and *wchA* present clear divergence patterns between the two groups. As expected, given their sero-specific origins, *wzx* and *wzy* present a significant number of alignment gaps. This contrasts the other genes, although some structure can be seen within serogroups for genes *wzx* and *wzy* (ex: serogroups 6 and 23).

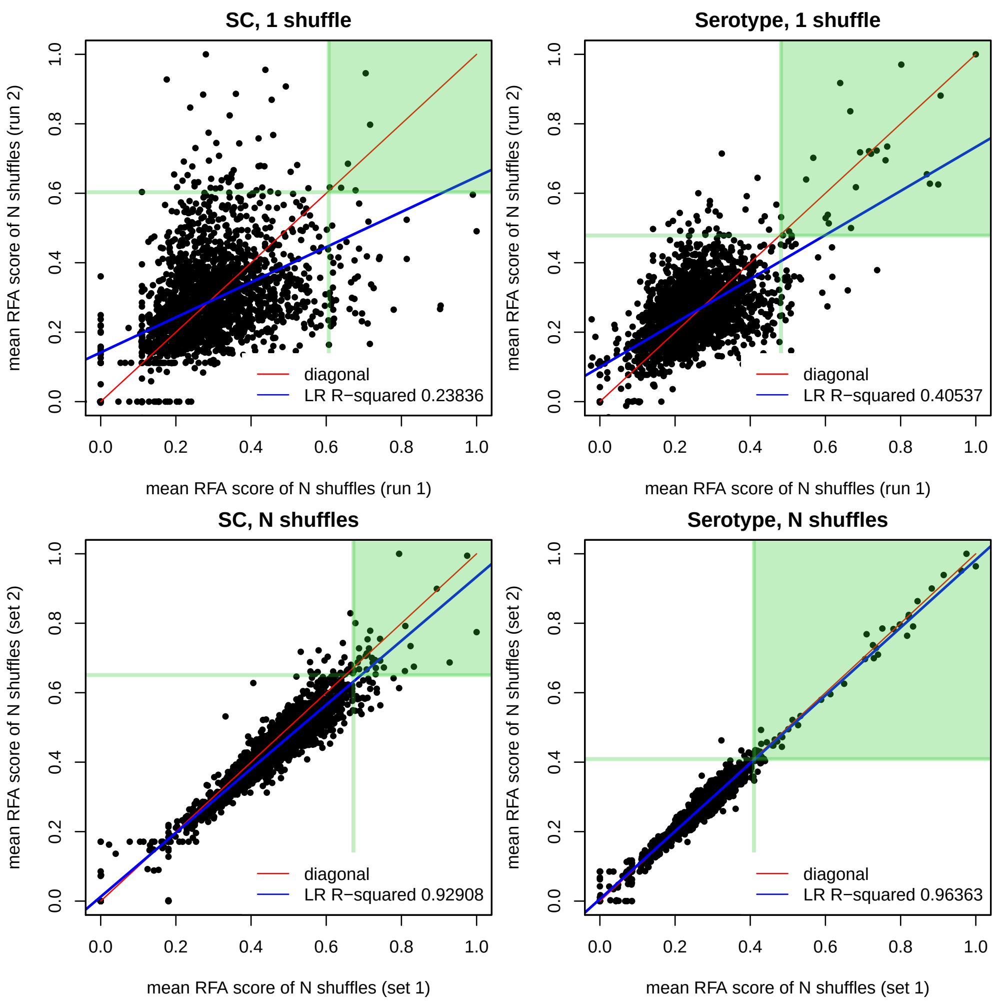

Figure 5: **Sensitivity analysis on numerical predictor assumption (without cutoff of gene mismatches).** In each subplot, the obtained RFA scores of each gene are compared between two experiments using independent inputs. The red line (diagonal) represents the expectation of gene scores being the same in two independent RFA experiments if the numerical assumption of predictor variables would not introduce RFA bias. The blue line is the linear regression line of gene scores (LR is linear-regression). *(top row)* Two RFAs are run on two independently randomized input matrices (see Methods description in the Main Text). The results show a low correlation between the scores obtained for the same genes in the two runs, is particular for SC classification. This demonstrates an existing bias in the RFA method depending on specific allelic numerations. *(bottom row)* RFAs are run on two independent sets of $N = 50$ randomized input matrices (see Methods description in the Main Text). Gene scores are averaged over the N RFAs for each set. The results show a very high correlation between the scores obtained for the same genes in the two input sets, specially for Serotype classification. This demonstrates that by averaging scores over N input randomized matrices, the existing RFA bias due to allelic numeration is mitigated. In particular, the variation of mean scores for the top 2.5% ranking sites (green shaded area) is shown to be small, specially for Serotype classification, such that the same genes are effectively selected on that upper limit in any set of N input matrices.

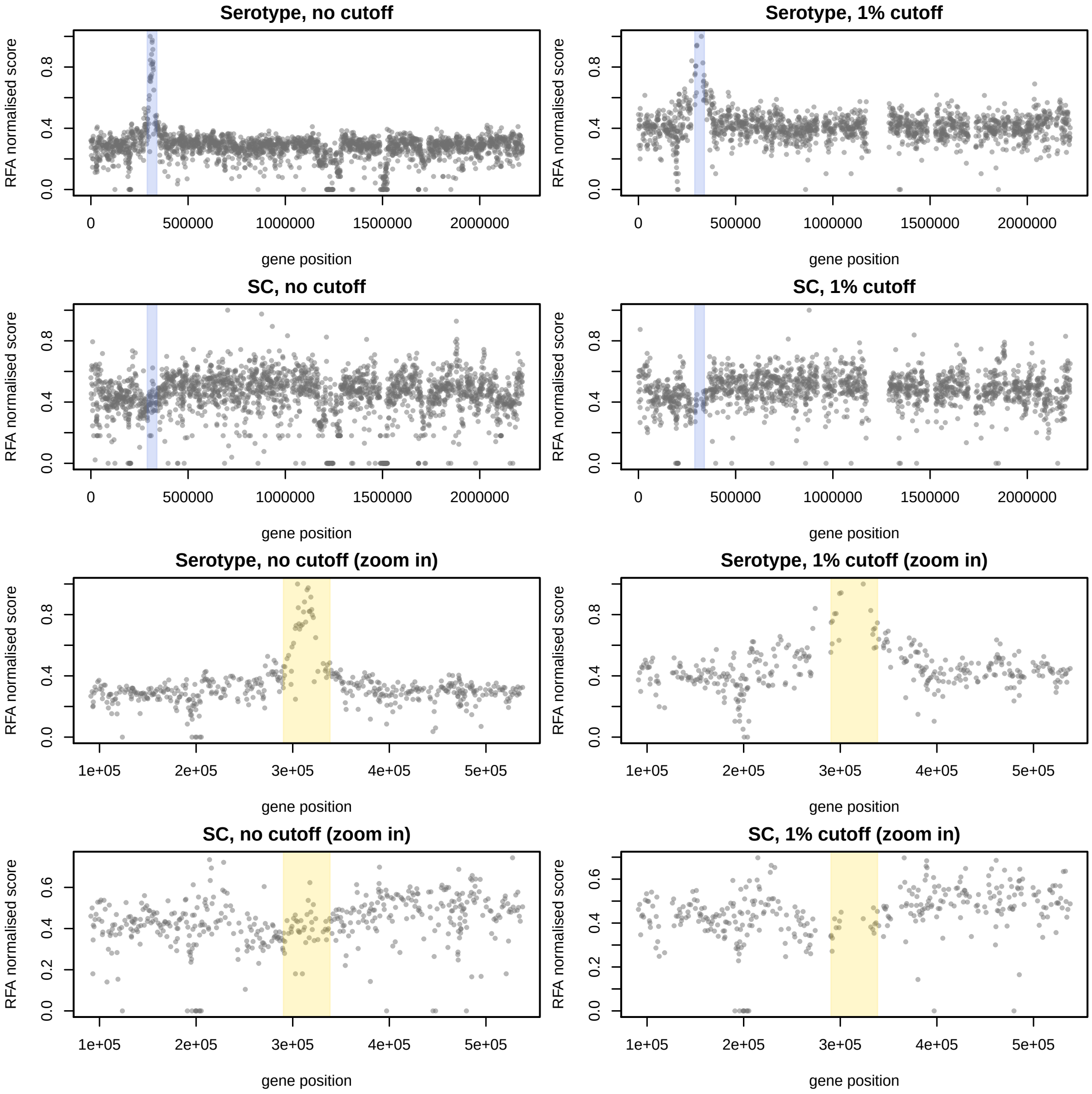

Figure 6: **RFA ranking versus gene position.** The RFA normalised scores for 4 scenarios are presented: Serotype and SC classification, with no cutoff *(left column)* and 1% cutoff *(right column)* for gene mismatches (1% cutoff as a rejection of genes with an excess of 99% of mismatches). For simplicity, we represent the circular genome in a linear form, with the first gene at position 186 being *dnaA* and the last gene at position 2220530 being *parB*. *(left column)* The genes with highest scores for serotype classification are seen to cluster around the capsular locus (blue area). The bottom two subplots, presenting a zoom in the capsular region show that core capsular genes (centre of yellow region) score highly, but flanking genes also present high scores with decreasing values with distance. *(right column)* Core genes from the capsular locus (centre of blue and yellow areas) are not considered, since these present an excess of 99% of mismatches to the reference genome. However, genes flanking the capsular locus still score highly for serotype classification and scores decrease with distance.

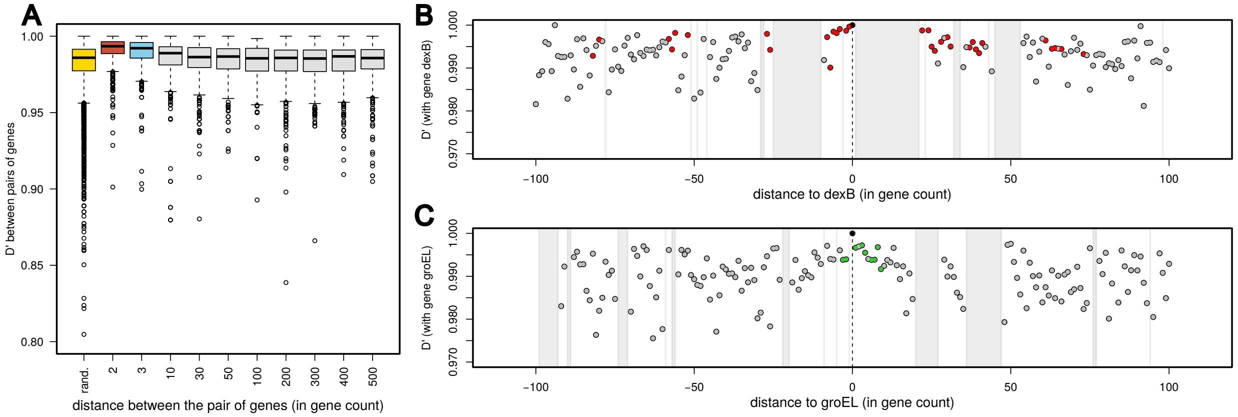

Figure 7: **Linkage disequilibrium.** *(A)* Distributions of linkage (D') for different selections (cassettes) of genes. For the randomly selected gene pairs ('rand', yellow), representing a genome sample of 20,000 random pairs, the median is 0.98, with 95% of the values $\in \{0.94, 0.99\}$ (as in Watkins et al). For the capsular locus ('CPS', red), 10 genes are considered upstream and downstream starting at genes aliA and dexB (as in the main text). For the groEL (blue) 10 genes are considered upstream and downstream of the gene groEL. For the remaining cassettes (grey), 10 genes are considered upstream and downstream of each named gene in the x-axis. *(B)* Linkage between the gene dexB and all other genes at a maximum distance of 100 genes. Red points are the genes of Table 1 (main text) that are 100 genes away from dexB. *(C)* Linkage between the gene groEL and all other genes at a maximum distance of 100 genes. Gree points are the genes of Table 2 (main text) that are 100 genes away from groEL. In *B,C* subplots, the vertical dashed lines mark the gene of reference (dexB or groEL). In *A,B,C* subplots, linkage is calculated according to Lewontin's normalization and excluding all genes which showed mismatches or deletions above a threshold of 1% (exclusions are marked with grey areas in subplots *B,C*).

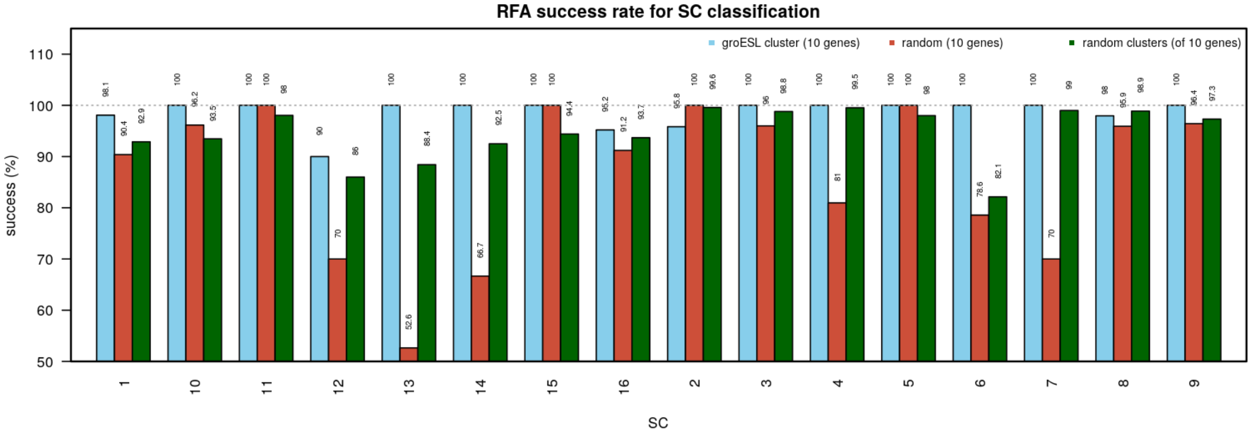

Figure 8: **Comparison of random forest SC classification success rates when using the *groESL* cluster of genes or random genes.** Results from experiments are presented in different colors, in blue when using the 10 genes highly informative for SC classification clustering around the *groESL* locus (see Figure 2, Table 2), in red when using 10 genes randomly selected from the genome, in green when using using random sets of 10 contiguous genes (presented is the mean classification for 10 RFA runs using 10 independent clusters of such 10 genes). The *groESL* cluster is seen to be the most informative (100% of the SCs are predicted with accuracy above or equal to 90%) when compared to 10 random genes (for which ≈ 43% of SCs have classification success lower than 90% and ≈ 31% have classification success lower than or equal to 70%), or for the mean classification of 10 contiguous genes (81% of the SCs are predicted with accuracy above or equal to 90%, and only 12% of SCs are better classified when compared to the results of the *groESL* cluster). Only genes with less than 1% of mismatches are included.

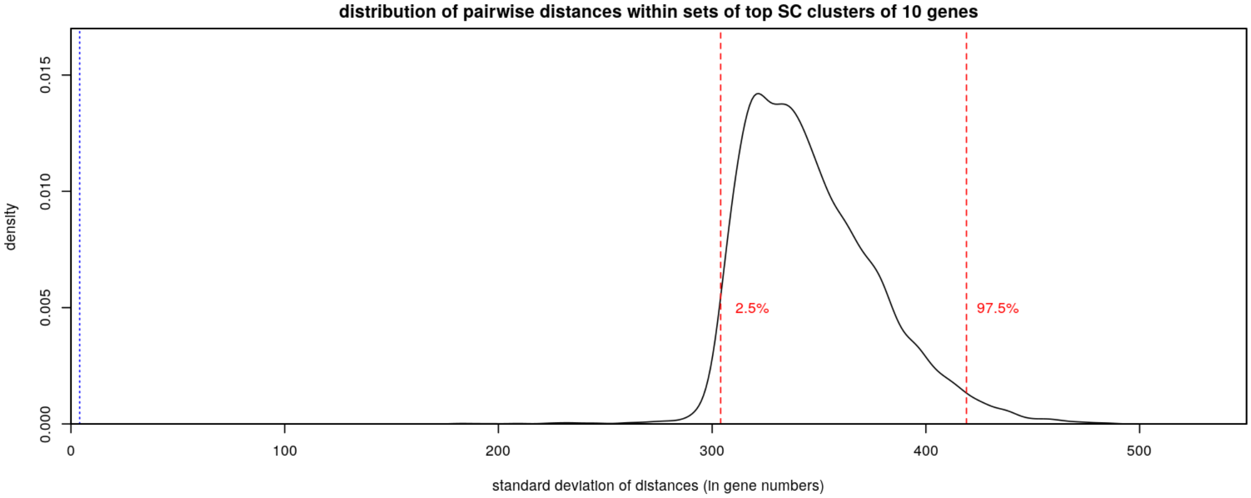

Figure 9: **Distribution of distances within clusters of 10 genes sampled from the top SC predictor genes.** The main results describe the unexpected result of a cluster of 10 genes within and around the *groESL* operon when classifying SC (genes shown in Figure 2, Table 2). To quantify the significance level of this finding, we here present the distribution of distances found among such top genes. From the 41 genes found as good predictors for SC (Table 2), we sample 10,000 independent sets of 10 genes. From each set, we use the each gene's genome position to calculate the standard-deviation (StDev) of the distance (number of genes) between every 2 genes in the set. Given the circular nature of the genome, we use the minimum possible distance. The resulting distribution is shown in the plot. The 95% boundaries are presented in red (dashed lines). The StDev of the distances found within the cluster of 10 genes including the *groESL* operon is shown in blue (dotted line). Finding such cluster of genes has a p-value of $\approx 1.52e-06$ and is therefore significant in the background of the observed distribution.

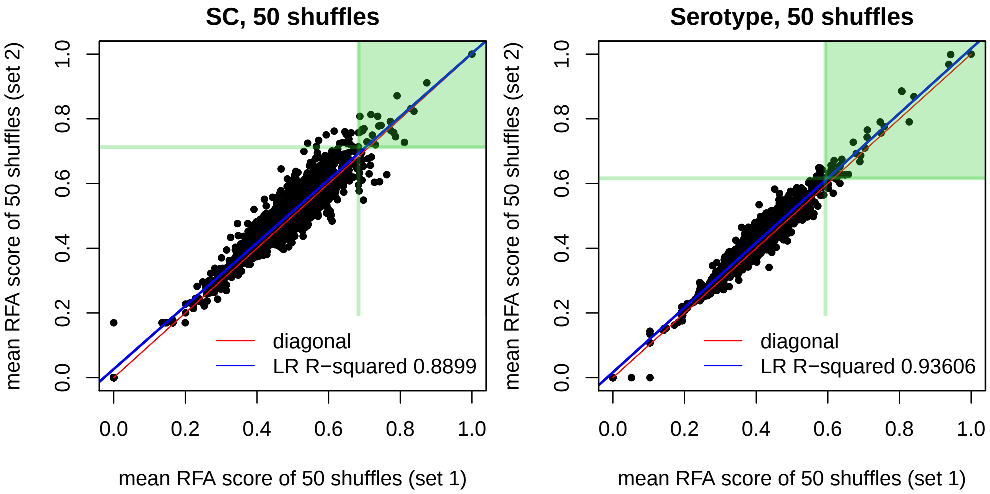

Figure 10: **Sensitivity analysis on numerical predictor assumption (1% cutoff of gene mismatches).** In each subplot, the obtained RFA scores of each gene are compared between two experiments using independent inputs. The red line (diagonal) represents the expectation of gene scores being the same in two independent RFA experiments if the numerical assumption of predictor variables would not introduce RFA bias. The blue line is the linear regression line of gene scores (LR is linear-regression). RFAs are run on two independent sets of $N = 50$ randomized input matrices (see Methods description in the Main Text). Gene scores are averaged over the N RFAs for each set. The results show a very high correlation between the scores obtained for the same genes in the two input sets. Only genes with less than 1% of mismatches are included. These plots demonstrate that by averaging scores over N input randomized matrices, the existing RFA bias due to allelic numeration is mitigated. In particular, the variation of mean scores for the top 2.5% ranking sites (green shaded area) is shown to be small (and smaller than when considering all genes, Fig S5), such that the same genes are effectively selected on that upper limit in any set of N input matrices. For the cluster of 10 genes around the *groESL* operon, found to be informative for SC in the results of the main text (Figure 2B, Table 2), 8 out of 10 were consistently found in the top 2.5% of both sets of $N = 50$. The exceptions were the genes SPN23F19240 (recX) and SPN23F19250.

References

Sirkka-Liisa Varvio, Kari Auranen, Elja Arjas, and P. Helena Mäkelä. Evolution of the Capsular Regulatory Genes in Streptococcus pneumoniae. The Journal of Infectious Diseases 2009; 200:1144–51. doi:10.1086/605651

Watkins, E. R., Penman, B. S., Lourenço, J., Buckee, C. O., Martin, C., Maiden, M. C. J., & Gupta, S. Vaccination Drives Changes in Metabolic and Virulence Profiles of Streptococcus pneumoniae. PLoS Pathogens 2015, 11(7), e1005034. doi:10.1371/journal.ppat.1005034

Supplementary Material - Gene function tables

Description of top-scoring genes highly informative for serotype classification

Gene	Function / Type	Description / Background
trpF	Amino acid biosynthesis	Known to be essential for the biosynthesis of tryptophan for *S. pneumoniae* [1], and more generally of the biosynthesis of aromatic amino acids in at least 9 species of bacteria [2].
fabG	Fatty acid biosynthesis	Encodes the beta-ketoacyl-ACP reductase, the only known keto-acid reductase in bacterial fatty acid biosynthesis [3].
lysC	Amino acid biosynthesis	Encodes for an aspartokinase involved in lysine production and aminoethyl cysteine resistance in *Corynebacterium glutamicum* [4].
mvaD<br>mvaK2	Cell wall biosynthesis<br><br>Electron transport<br><br>Aerobic cellular respiration	Involved in the Mevalonate pathway, also known as the HMG-CoA reductase pathway, found in bacteria, eukaryotes and archaea [5]. One of the pathway's main products, the isopentenyl pyrophosphate (IPP), is used to make isoprenoids, a diverse class of over 30,000 biomolecules. In bacteria, the principal products of IPP include the lipid carrier undecaprenol (involved in wall biosynthesis), plus a range of menaquinones and ubiquinones both involved in electron transport, and the latter also in aerobic cellular respiration [6-8]. In *S. pneumoniae*, these two genes are essential for growth and are proposed to be part of a single operon [6].
spuA	Glycogen metabolism	The SpuA protein is involved in alpha-glucan metabolism, whose main substrate is glycogen (polysaccharide of glucose), an abundant resource in human lung epithelial cells [9-10]. The protein SpuA has also been shown to be highly immunogenic [11].
patB	ATP-binding cassette (ABC) transporter<br><br>Resistance to fluoroquinolones	Encodes part of an ABC efflux pump, responsible for resistance to fluoroquinolones [12-14].
pitA<br>pitB	ATP-binding cassette (ABC) transporter<br><br>Iron uptake	These genes were located within the *pit* operon, encoding for an ABC transporter involved in iron uptake. In line with our findings, the *pit* operon has previously been shown to exhibit strain-specific variation [15].
gnd	Iron uptake	Regulator of iron transport [16]. *gnd* is transcriptionally linked to the top-scoring gene *ritR*.
ritR	Capsular biosynthesis	*ritR* is orthologous to the streptococcal global regulator *covR*, for which there is conclusive evidence from *S. pyogenes*, *S. suis* and *S. agalactiae* of regulatory functions on capsular biosynthesis [17-19]. *ritR* is transcriptionally linked to the top-scoring gene *gnd*.
ecsA	ATP-binding	One of two genes that encode for the Ecs ABC transporter. The substrate of Ecs

	cassette (ABC) transporter<br><br>Aerobic cellular respiration	is so far unknown, but obligatory anaerobes or microaerophilic bacteria do not carry the Ecs transporter, and its function is therefore argued to be related to respiration [20].
glyP	Symporter<br><br>Sodium uptake	Transport can be achieved by a multitude of systems alternative to ABC transporters, such as 'passive' channels like the sodium symporter GlyP, encoded by the gene *glyP*. Sodium is one of the main electrolytes in human saliva, existing there at a higher concentration than in blood plasma, and differentiation in sodium transport, similarly to iron or glucose transport, could potentially be under selection for niche specialization [21].
pbp1A<br><br>pbpX<br><br>mraW<br><br>mraY	Cell wall biosynthesis<br><br>Penicillin resistance<br><br>beta-lactam resistance<br><br>Cell division	Genes flanking the capsular locus involved in the cell wall biosynthesis pathway [22]. Mutations in these genes can lead to penicillin resistance, and single-nucleotide positions associate strongly with *S. pneumoniae* beta-lactam resistance [23-25]. In *S. pneumoniae*, *pbp1A* is also involved in the formation of the septum during cell division [26]. *pbp1A* is associated in a two-gene operon with another top-scoring gene, *recU* [27-28].
vraT<br><br>vraS	Resistance tomethicillin, vancomycin, daptomycin	Resistance to various classes of cell wall-inhibitory antibiotics (ex. methicillin, vancomycin, daptomycin) in *S. Aureus* is regulated via the *vra* operon, by up or downregulation of a set of genes commonly designated as the cell wall stimulon [29]. This operon is encoded the *vraT* and *vraT* genes.
blpH	Bacteriocin production	Part of the BlpABCSRH pathway [30], which regulates production of class II bacteriocins and related immunity proteins [31-32].
glmS	Production of ammonia	In related species, the aminotransferase GlmS is known to upregulate the production of ammonia thereby increasing acid tolerance and survival [33].
recU	DNA recombination, repair and segregation<br><br>Cell division	Encodes the Holliday junction resolvase, required for homologous DNA recombination, repair and chromosome segregation [27-28]. *recU* is associated in a two-gene operon with another top-scoring gene, *pbp1A* [27-28].
luxS	Biofilm formation<br><br>Pneumolysin expression	The capsular flanking gene *luxS* is part of a *Staphylococcus epidermidis* quorum-sensing system in biofilm formation, and linked to pneumolysin expression, a key player in interference with the host immune response [34-35].
lytC	Lysozyme production	Encodes a lysozyme (or glycoside hydrolase) which can be found in a number of secretions, such as tears, saliva and mucus, with the potential to damage (interspecies) bacterial cell walls by catalyzing hydrolysis of linkages and residues in peptidoglycans and chitodextrins [36-37].

Description of top-scoring genes highly informative for SC classification

Gene	Function / Type	Description / Background
sodA	Survival<br><br>Virulence	Encodes for the manganese superoxide dismutase, critical against oxidative stress and linked to both survival and virulence; has been highlighted in numerous studies for its relevance in identification of rare clones of pneumococci [38-39] and Streptococci at the species level [40-41].
lmb	Virulence<br><br>Laminin binding	Encodes for an extracellular protein (Lmb) with a key role in physiology and pathogenicity [42-43], and homologs of this protein have been documented to be present and discriminatory of at least 25 groups of the *Streptococcus* genus with possible similar functions [44-45]. The Lmb protein also called laminin-binding protein, given its capacity to bind to laminin present in the host's extracellular matrix and is immunogenic [45].
pdhB	Virulence<br><br>Degradation of fibrinogen<br><br>Fibronectin binding	Glycolytic enzymes (GE) such as the one encoded by *pdhB* have long been regarded as virulence factors [46] and are involved in cytosol-located metabolic processes. When transported to the surface, the PdhB protein-complex is known to interact with host factors such as the extracellular matrix and fibrinolysis system [47]. Critically, *Mycoplasma pneumoniae*'s *pdhB* is involved in the degradation of human fibrinogen and is also able to bind human fibronectin [47-48]. Fibronectin is commonly found in human saliva, presenting a vast set of functions, from prevention to colonization of the oral cavity and pharynx, to involvement in adhesion and wound healing [49].
pclA	Host-cell adherence and invasion<br><br>Collagen mimicry	Encodes for the pneumococcal collagen-like protein A, a top candidate for human collagen mimicry [50], involved in host-cell adherence and invasion \cite{Paterson2008}. Binding to fibronectin and collagen are common strategies employed by various invading bacterial pathogens to colonize or disseminate within the host [51-52].
mreD	Cell shape	In ovococcus bacteria such as *S. pneumoniae* the function of the top-scoring protein MreD (the Rod shape-determining protein) is unknown. Depletion of MreD protein can cause cells to stop growing, become spherical, form chains and lyse [53].
SPN23F11320<br><br>SPN23F09460	Acetylation	The genes designated as SPN23F11320 and SPN23F09460 represent about 13\% of all non-putative GCN5-related, N-acetyltransferases of the (GNAT) family present in our dataset. These are key proteins involved in acetylation, and there is growing evidence in the literature of their role in regulation of central carbon metabolism and phenotype through epigenetics [54-55].
recX	Regulation of recombination	The gene *recX* is in close proximity to the *groESL* operon, which encodes a regulatory protein that inhibits the RecA recombinase in multiple species of bacteria [56-59]. Its immediate function is to regulate recombination.

groEL<br><br>groES	Protein folding	Encode a chaperonin system for protein folding [60]. Apart from assisting protein folding by preventing inappropriate interactions between non-native polypeptides [60], this system may also buffer deleterious effects of mutations on protein foldability and stability [61]. The protein GroEL is highly immunogenic for different bacterial species and has been shown to provide strain-specific protection in vaccine studies [62-64]. Other studies have reported the power of the *groESL* operon and its proteins to ascertain phylogeny and classification within the *{Streptococcus* genus [65] and between species of the *Viridans* and *Mutans* Streptococci groups [66-67].
carB	Production of O-antigen	Many bacterial species use O-antigen to avoid phagocytosis and to resist the lytic action of the complement system [68]. For Salmonella typhimurium it is known that o-antigen's immunogenicity is species dependent [69]. In *E. coli*, over 160 different o-antigen structures are known to be produced and are strain dependent [70].
vanZ	Virulence<br><br>Resistance to teicoplanin	The protein VanZ is linked to teicoplanin resistance in *Enterococcus faecium* [71] and is also essential for lung infection in *S. pneumoniae* [72].
licA	Virulence<br><br>Modulation of PCHO	LicA encodes the enzyme phosphorylcholine kinase which catalyses the incorporation of phosphorylcholine (PCHO) into surface components [73]. PCHO is a plurispecific vaccine candidate, since it is present on the surface of many mucosal organisms including *Haemophilus influenzae*, *Streptococcus pneumoniae* and *Pseudomonas aeruginosa*, and in the pili of *Neisseria meningitidis* and *Neisseria gonorrhoeae*. Furthermore, modulation of PCHO expression has the potential to confer the property of immune avoidance and persistence on mucosa by Neisseria species [74] and pathogenesis of invasive disease by *Haemophilus influenzae* type b [73].

References:

[1] Jurgens, C., Strom, A., Wegener, D., Hettwer, S., Wilmanns, M., & Sterner, R. (2000). Directed evolution of a (alpha-beta)8-barrel enzyme to catalyze related reactions in two different metabolic pathways. Proceedings of the National Academy of Sciences of the United States of America, 97(18), 9925–9930. http://doi.org/http://dx.doi.org/10.1073/pnas.160255397

[2] Panina, E. M., Vitreschak, A. G., Mironov, A. A., & Gelfand, M. S. (2003). Regulation of biosynthesis and transport of aromatic amino acids in low-GC Gram-positive bacteria. FEMS Microbiology Letters, 222(2), 211–220. http://doi.org/10.1016/S0378-1097(03)00303-3

[3] Patel, M. P., Liu, W. S., West, J., Tew, D., Meek, T. D., & Thrall, S. H. (2005). Kinetic and chemical mechanisms of the fabG-encoded Streptococcus pneumoniae β-ketoacyl-ACP reductase.

Biochemistry, 44(50), 16753–16765. http://doi.org/10.1021/bi050947j

[4] Kalinowski, J., Bachmann, B., Thierbach, G., & Pühler, A. (1990). Aspartokinase genes lysC alpha and lysC beta overlap and are adjacent to the aspartate beta-semialdehyde dehydrogenase gene asd in Corynebacterium glutamicum. Molecular & General Genetics : MGG, 224(3), 317–24. Retrieved from http://www.ncbi.nlm.nih.gov/pubmed/1980002

[5] Buhaescu, I., & Izzedine, H. (2007). Mevalonate pathway: A review of clinical and therapeutical implications. Clinical Biochemistry, 40(9-10), 575–584. http://doi.org/10.1016/j.clinbiochem.2007.03.016

[6] Wilding, E. I., Brown, J. R., Bryant, A. P., Chalker, A. F., Holmes, D. J., Ingraham, K. A., … So, C. H. I. Y. (2000). Identification , Evolution , and Essentiality of the Mevalonate Pathway for Isopentenyl Diphosphate Biosynthesis in Gram-Positive Cocci, 182(15), 4319–4327. http://doi.org/10.1128/JB.182.15.4319-4327.2000.Updated

[7] Buhaescu, I., & Izzedine, H. (2007). Mevalonate pathway: A review of clinical and therapeutical implications. Clinical Biochemistry, 40(9-10), 575–584. http://doi.org/10.1016/j.clinbiochem.2007.03.016

[8] Holstein, S. A., & Hohl, R. J. (2004). Isoprenoids: remarkable diversity of form and function. Lipids, 39(4), 293–309. Retrieved from http://www.ncbi.nlm.nih.gov/pubmed/15357017

[9] van Bueren, A. L., Higgins, M., Wang, D., Burke, R. D., & Boraston, A. B. (2007). Identification and structural basis of binding to host lung glycogen by streptococcal virulence factors. Nature Structural & Molecular Biology, 14(1), 76–84. http://doi.org/10.1038/nsmb1187

[10] Abbott, D. W., Higgins, M. A., Hyrnuik, S., Pluvinage, B., Lammerts van Bueren, A., & Boraston, A. B. (2010). The molecular basis of glycogen breakdown and transport in Streptococcus pneumoniae. Molecular Microbiology, 77(1), 183–199. http://doi.org/10.1111/j.1365-2958.2010.07199.x

[11] Bongaerts, R. J., Heinz, H. P., Hadding, U., & Zysk, G. (2000). Antigenicity, expression, and molecular characterization of surface- located pullulanase of Streptococcus pneumoniae. Infect.Immun., 68(12), 7141–7143. http://doi.org/Doi 10.1128/Iai.68.12.7141-7143.2000

[12] Garvey, M. I., Baylay, A. J., Wong, R. L., & Piddock, L. J. V. (2011). Overexpression of patA and patB, which encode ABC transporters, is associated with fluoroquinolone resistance in clinical isolates of Streptococcus pneumoniae. Antimicrobial Agents and Chemotherapy, 55(1), 190–196. http://doi.org/10.1128/AAC.00672-10

[13] El Garch, F., Lismond, A., Piddock, L. J. V, Courvalin, P., Tulkens, P. M., & van Bambeke, F. (2010). Fluoroquinolones induce the expression of patA and patB, which encode ABC efflux pumps in Streptococcus pneumoniae. Journal of Antimicrobial Chemotherapy, 65(10),

2076–2082. http://doi.org/10.1093/jac/dkq287

[14] Boncoeur, E., Durmort, C., Bernay, B., Ebel, C., Di Guilmi, A. M., Croizé, J., … Jault, J. M. (2012). PatA and PatB form a functional heterodimeric ABC multidrug efflux transporter responsible for the resistance of streptococcus pneumoniae to fluoroquinolones. Biochemistry, 51(39), 7755–7765. http://doi.org/10.1021/bi300762p

[15] Jomaa, M., Terry, S., Hale, C., Jones, C., Dougan, G., & Brown, J. (2006). Immunization with the iron uptake ABC transporter proteins PiaA and PiuA prevents respiratory infection with Streptococcus pneumoniae. Vaccine, 24(24), 5133–5139. http://doi.org/10.1016/j.vaccine.2006.04.012

[16] Ulijasz, A. T., Andes, D. R., Glasner, J. D., & Weisblum, B. (2004). Regulation of Iron Transport in Streptococcus pneumoniae by RitR , an Orphan Response Regulator Regulation of Iron Transport in Streptococcus pneumoniae by RitR , an Orphan Response Regulator. Journal of Bacteriology, 186(23), 8123–8136. http://doi.org/10.1128/JB.186.23.8123

[17] Graham, M. R., Smoot, L. M., Migliaccio, C. A. L., Virtaneva, K., Sturdevant, D. E., Porcella, S. F., … Musser, J. M. (2002). Virulence control in group A Streptococcus by a two-component gene regulatory system: global expression profiling and in vivo infection modeling. Proceedings of the National Academy of Sciences of the United States of America, 99(21), 13855–60. http://doi.org/10.1073/pnas.202353699

[18] Lamy, M.-C., Zouine, M., Fert, J., Vergassola, M., Couve, E., Pellegrini, E., … Poyart, C. (2004). CovS/CovR of group B streptococcus: a two-component global regulatory system involved in virulence. Molecular Microbiology, 54(5), 1250–68. http://doi.org/10.1111/j.1365-2958.2004.04365.x

[19] Pan, X., Ge, J., Li, M., Wu, B., Wang, C., Wang, J., … Tang, J. (2009). The orphan response regulator CovR: a globally negative modulator of virulence in Streptococcus suis serotype 2. Journal of Bacteriology, 191(8), 2601–12. http://doi.org/10.1128/JB.01309-08

[20] Jonsson, I. M., Juuti, J. T., François, P., AlMajidi, R., Pietiäinen, M., Girard, M., … Kontinen, V. P. (2010). Inactivation of the Ecs ABC transporter of Staphylococcus aureus attenuates virulence by altering composition and function of bacterial wall. PLoS ONE, 5(12). http://doi.org/10.1371/journal.pone.0014209

[21] Reizer, J., Reizer, A., & Saier, M. H. (1994). A functional superfamily of sodium/solute symporters. Biochimica et Biophysica Acta (BBA) - Reviews on Biomembranes, 1197(2), 133–166. http://doi.org/10.1016/0304-4157(94)90003-5

[22] Wen, Z., Liu, Y., Qu, F., & Zhang, J.-R. (2016). Allelic Variation of the Capsule Promoter Diversifies Encapsulation and Virulence In Streptococcus pneumoniae. Scientific Reports, 6, 30176. http://doi.org/10.1038/srep30176

[23] Croucher, N. J., Finkelstein, J. A., Pelton, S. I., Mitchell, P. K., Lee, G. M., Parkhill, J., … Lipsitch, M. (2013). Population genomics of post-vaccine changes in pneumococcal epidemiology. Nature Genetics, 45(6), 656–63. http://doi.org/10.1038/ng.2625

[24] Chewapreecha, C., Marttinen, P., Croucher, N. J., Salter, S. J., Harris, S. R., Mather, A. E., … Parkhill, J. (2014). Comprehensive Identification of Single Nucleotide Polymorphisms Associated with Beta-lactam Resistance within Pneumococcal Mosaic Genes. PLoS Genetics, 10(8). http://doi.org/10.1371/journal.pgen.1004547

[25] Pillai, D. R., Shahinas, D., Buzina, A., Pollock, R. A., Lau, R., Khairnar, K., … Low, D. E. (2009). Genome-wide dissection of globally emergent multi-drug resistant serotype 19A Streptococcus pneumoniae. BMC Genomics, 10, 642. http://doi.org/10.1186/1471-2164-10-642

[26] Paik, J., Kern, I., Lurz, R., & Hakenbeck, R. (1999). Mutational analysis of the Streptococcus pneumoniae bimodular class A penicillin-binding proteins. Journal of Bacteriology, 181(12), 3852–3856.

[27] Morlot, C., Zapun, A., Dideberg, O., & Vernet, T. (2003). Growth and division of Streptococcus pneumoniae: localization of the high molecular weight penicillin-binding proteins during the cell cycle. Molecular Microbiology, 50(3), 845–55. Retrieved from http://www.ncbi.nlm.nih.gov/pubmed/14617146

[28] Pedersen, L. B., & Setlow, P. (2000). Penicillin-binding protein-related factor A is required for proper chromosome segregation in Bacillus subtilis. Journal of Bacteriology, 182(6), 1650–8. Retrieved from http://www.ncbi.nlm.nih.gov/pubmed/10692371

[29] Boyle-Vavra, S., Yin, S., Jo, D. S., Montgomery, C. P., & Daum, R. S. (2013). VraT/YvqF is required for methicillin resistance and activation of the VraSR regulon in Staphylococcus aureus. Antimicrobial Agents and Chemotherapy, 57(1), 83–95. http://doi.org/10.1128/AAC.01651-12

[30] Knutsen, E., Ween, O., & Håvarstein, L. S. (2004). Two Separate Quorum-Sensing Systems Upregulate Transcription of the Same ABC Transporter in Streptococcus pneumoniae. Journal of Bacteriology, 186, 3078–3085. http://doi.org/10.1128/JB.186.10.3078-3085.2004

[31] De Saizieu, A., Gardes, C., Flint, N., Wagner, C., Kamber, M., Mitchell, T. J., … Lange, R. (2000). Microarray-based identification of a novel Streptococcus pneumoniae regulon controlled by an autoinduced peptide. Journal of Bacteriology, 182(17), 4696–4703. http://doi.org/10.1128/JB.182.17.4696-4703.2000

[32] Reichmann, P., & Hakenbeck, R. (2000). Allelic variation in a peptide-inducible two-component system of Streptococcus pneumoniae. FEMS Microbiology Letters, 190, 231–236. http://doi.org/10.1111/j.1574-6968.2000.tb09291.x

[33] Moye, Z. D., Burne, R. A., & Zeng, L. (2014). Uptake and metabolism of N-acetylglucosamine and glucosamine by Streptococcus mutans. Applied and Environmental Microbiology, 80(16), 5053–5067. http://doi.org/10.1128/AEM.00820-14

[34] Joyce, E. A., Kawale, A., Censini, S., Kim, C. C., Covacci, A., & Falkow, S. (2004). LuxS Is Required for Persistent Pneumococcal Carriage and Expression of Virulence and Biosynthesis Genes. Infection and Immunity, 72(5), 2964–2975. http://doi.org/10.1128/IAI.72.5.2964-2975.2004

[35] Xu, L., Li, H., Vuong, C., Vadyvaloo, V., Wang, J., Yao, Y., … Gao, Q. (2006). Role of the luxS Quorum-Sensing System in Biofilm Formation and Virulence of Staphylococcus epidermidis Role of the luxS Quorum-Sensing System in Biofilm Formation and Virulence of Staphylococcus epidermidis. Infection and Immunity, 74(1), 488–496. http://doi.org/10.1128/IAI.74.1.488

[36] García, P., González, M. P., García, E., García, J. L., & López, R. (1999). The molecular characterization of the first autolytic lysozyme of Streptococcus pneumoniae reveals evolutionary mobile domains. Molecular Microbiology, 33(1), 128–138. http://doi.org/10.1046/j.1365-2958.1999.01455.x

[37] Eldholm, V., Johnsborg, O., Haugen, K., Ohnstad, H. S., & Havastein, L. S. (2009). Fratricide in Streptococcus pneumoniae: Contributions and role of the cell wall hydrolases CbpD, LytA and LytC. Microbiology, 155(7), 2223–2234. http://doi.org/10.1099/mic.0.026328-0

[38] Obregón, V., García, P., García, E., Fenoll, A., López, R., & García, J. L. (2002). Molecular peculiarities of the lytA gene isolated from clinical pneumococcal strains that are bile insoluble. Journal of Clinical Microbiology, 40(7), 2545–2554. http://doi.org/10.1128/JCM.40.7.2545-2554.2002

[39] Arbique, J. C., Poyart, C., Trieu-Cuot, P., Quesne, G., Carvalho, M. D. G. S., Steigerwalt, A. G., … Facklam, R. R. (2004). Accuracy of phenotypic and genotypic testing for identification of Streptococcus pneumoniae and description of Streptococcus pseudopneumoniae sp. nov. Journal of Clinical Microbiology, 42(10), 4686–4696. http://doi.org/10.1128/JCM.42.10.4686-4696.2004

[40] Poyart, C., Quesne, G., Coulon, S., Berche, P., & Trieu-Cuot, P. (1998). Identification of streptococci to species level by sequencing the gene encoding the manganese-dependent superoxide dismutase. Journal of Clinical Microbiology, 36, 41–47.

[41] Martín-Galiano, A. J., Balsalobre, L., Fenoll, A., & De la Campa, A. G. (2003). Genetic characterization of optochin-susceptible viridans group streptococci. Antimicrobial Agents and Chemotherapy, 47, 3187–3194. http://doi.org/10.1128/AAC.47.10.3187-3194.2003

[42] Spellerberg, B., Rozdzinski, E., Martin, S., Weber-Heynemann, J., Schnitzler, N., Lütticken, R., & Podbielski, A. (1999). Lmb, a protein with similarities to the LraI adhesin family, mediates

attachment of Streptococcus agalactiae to human laminin. Infection and Immunity, 67(2), 871–8. Retrieved from http://www.ncbi.nlm.nih.gov/pubmed/9916102

[43] Terao, Y., Kawabata, S., Kunitomo, E., Nakagawa, I., & Hamada, S. (2002). Novel laminin-binding protein of Streptococcus pyogenes, Lbp, is involved in adhesion to epithelial cells. Infection and Immunity, 70(2), 993–997. http://doi.org/10.1128/IAI.70.2.993

[44] Zhang, Y. M., Shao, Z. Q., Wang, J., Wang, L., Li, X., Wang, C., … Pan, X. (2014). Prevalent distribution and conservation of streptococcus suis lmb protein and its protective capacity against the chinese highly virulent strain infection. Microbiological Research, 169, 395–401. http://doi.org/10.1016/j.micres.2013.09.007

[45] Wahid, R. M., Yoshinaga, M., Nishi, J., Maeno, N., Sarantuya, J., Ohkawa, T., … Miyata, K. (2005). Immune response to a laminin-binding protein (Lmb) in group a streptococcal infection. Pediatrics International, 47(2), 196–202. http://doi.org/10.1111/j.1442-200x.2005.02038.x

[46] Pancholi, V., & Chhatwal, G. S. (2003). Housekeeping enzymes as virulence factors for pathogens. International Journal of Medical Microbiology : IJMM, 293(6), 391–401. http://doi.org/10.1078/1438-4221-00283

[47] Gründel, A., Pfeiffer, M., Jacobs, E., & Dumke, R. (2016). Network of surface-displayed glycolytic enzymes in Mycoplasma pneumoniae and their interactions with human plasminogen. Infection and Immunity, 84(3), 666–676. http://doi.org/10.1128/IAI.01071-15

[48] Dallo, S. F., Kannan, T. R., Blaylock, M. W., & Baseman, J. B. (2002). Elongation factor Tu and E1 beta subunit of pyruvate dehydrogenase complex act as fibronectin binding proteins in Mycoplasma pneumoniae. Molecular Microbiology, 46(4), 1041–51. Retrieved from http://www.ncbi.nlm.nih.gov/pubmed/12421310

[49] Pankov, R., & Yamada, K. M. (2002). Fibronectin at a glance. Journal of Cell Science, 115(Pt 20), 3861–3. Retrieved from http://www.ncbi.nlm.nih.gov/pubmed/12244123

[50] Doxey, A. C., & McConkey, B. J. (2013). Prediction of molecular mimicry candidates in human pathogenic bacteria. Virulence, 4, 453–466. http://doi.org/10.4161/viru.25180

[51] Eberhard, T., Virkola, R., Korhonen, T., Kronvall, G., & Ullberg, M. (1998). Binding to Human Extracellular Matrix by Neisseria meningitidis. Infection and Immunity, 66(4), 1791–1794.

[52] Agarwal, V., Kuchipudi, A., Fulde, M., Riesbeck, K., Bergmann, S., & Blom, A. M. (2013). Streptococcus pneumoniae Endopeptidase O (PepO) is a multifunctional plasminogen-and fibronectin-binding protein, facilitating evasion of innate immunity and invasion of host cells. Journal of Biological Chemistry, 288(10), 6849–6863. http://doi.org/10.1074/jbc.M112.405530

[53] Land, A. D., & Winkler, M. E. (2011). The requirement for pneumococcal MreC and MreD is

relieved by inactivation of the gene encoding PBP1a. Journal of Bacteriology, 193, 4166–4179. http://doi.org/10.1128/JB.05245-11

[54] Li, J., Li, J. W., Feng, Z., Wang, J., An, H., Liu, Y., … Zhang, J. R. (2016). Epigenetic Switch Driven by DNA Inversions Dictates Phase Variation in Streptococcus pneumoniae. PLoS Pathogens, 12(7), 1–36. http://doi.org/10.1371/journal.ppat.1005762

[55] Favrot, L., Blanchard, J. S., & Vergnolle, O. (2016). Bacterial GCN5-Related N -Acetyltransferases: From Resistance to Regulation. Biochemistry, 55(7), 989–1002. http://doi.org/10.1021/acs.biochem.5b01269

[56] Bergé, M., Mortier-Barrière, I., Martin, B., & Claverys, J. P. (2003). Transformation of Streptococcus pneumoniae relies on DprA- and RecA-dependent protection of incoming DNA single strands. Molecular Microbiology, 50(2), 527–536. http://doi.org/10.1046/j.1365-2958.2003.03702.x

[57] Venkatesh, R., Ganesh, N., Guhan, N., Reddy, M. S., Chandrasekhar, T., & Muniyappa, K. (2002). RecX protein abrogates ATP hydrolysis and strand exchange promoted by RecA: insights into negative regulation of homologous recombination. Proceedings of the National Academy of Sciences of the United States of America, 99(19), 12091–12096. http://doi.org/10.1073/pnas.192178999

[58] Stohl, E. A., Brockman, J. P., Burkle, K. L., Morimatsu, K., Kowalczykowski, S. C., & Seifert, H. S. (2003). Escherichia coli RecX inhibits RecA recombinase and coprotease activities in vitro and in vivo. Journal of Biological Chemistry, 278(4), 2278–2285. http://doi.org/10.1074/jbc.M210496200

[59] Galvão, C. W., Souza, E. M., Etto, R. M., Pedrosa, F. O., Chubatsu, L. S., Yates, M. G., … Steffens, M. B. R. (2012). The RecX protein interacts with the RecA protein and modulates its activity in herbaspirillum seropedicae. Brazilian Journal of Medical and Biological Research, 45(12), 1127–1134. http://doi.org/10.1590/S0100-879X2012007500160

[60] Hayer-Hartl, M., Bracher, A., & Hartl, F. U. (2016). The GroEL-GroES Chaperonin Machine: A Nano-Cage for Protein Folding. Trends in Biochemical Sciences. Elsevier Ltd. http://doi.org/10.1016/j.tibs.2015.07.009

[61] Williams, T. A., & Fares, M. A. (2010). The effect of chaperonin buffering on protein evolution. Genome Biology and Evolution, 2(1), 609–619. http://doi.org/10.1093/gbe/evq045

[62] Kim, S. N., Kim, S. W., Pyo, S. N., & Rhee, D. K. (2001). Molecular cloning and characterization of groESL operon in Streptococcus pneumoniae. Mol Cells, 11, 360–368.

[63] Cao, J., Zhang, X., Gong, Y., Zhang, Y., Cui, Y., Lai, X., … Zhang, L. (2013). Protection against pneumococcal infection elicited by immunization with multiple pneumococcal heat shock

proteins. Vaccine, 31(35), 3564–3571. http://doi.org/10.1016/j.vaccine.2013.05.061

[64] Péchiné, S., Hennequin, C., Boursier, C., Hoys, S., & Collignon, A. (2013). Immunization using GroEL decreases Clostridium difficile intestinal colonization. PLoS ONE, 8(11). http://doi.org/10.1371/journal.pone.0081112

[65] Glazunova, O. O., Raoult, D., & Roux, V. (2009). Partial sequence comparison of the rpoB, sodA, groEL and gyrB genes within the genus Streptococcus. International Journal of Systematic and Evolutionary Microbiology, 59, 2317–2322. http://doi.org/10.1099/ijs.0.005488-0

[66] Teng, L., Hsueh, P., Tsai, J., Hsu, J., Lai, H., Lee, C., … Ho, S. (2002). groESL Sequence Determination , Phylogenetic Analysis , and Species Differentiation for Viridans Group Streptococci groESL Sequence Determination , Phylogenetic Analysis , and Species Differentiation for Viridans Group Streptococci. Journal of Clinical Microbiology, 40, 3172–3178. http://doi.org/10.1128/JCM.40.9.3172

[67] Hung, W. C., Tsai, J. C., Hsueh, P. R., Chia, J. S., & Teng, L. J. (2005). Species identification of mutans streptococci by groESL gene sequence. Journal of Medical Microbiology, 54, 857–862. http://doi.org/10.1099/jmm.0.46180-0

[68] Kalynych, S., Morona, R., & Cygler, M. (2014). Progress in understanding the assembly process of bacterial O-antigen. FEMS Microbiology Reviews, 38(5), 1048–1065. http://doi.org/10.1111/1574-6976.12070

[69] Svenson, S. B., & Lindberg, A. L. F. A. (1981). Artificial Salmonella Vaccines : Salmonella typhimurium 0- Antigen-Specific Oligosaccharide-Protein Conjugates Elicit Protective Antibodies in Rabbits and Mice, 32(2), 490–496.

[70] Raetz, C. R., & Whitfield, C. (2002). Lipopolysaccharide endotoxins. Annual Review of Biochemistry, 71, 635–700. http://doi.org/10.1146/annurev.biochem.71.110601.135414

[71] Arthur, M., Depardieu, F., Molinas, C., Reynolds, P., & Courvalin, P. (1995). The vanZ gene of Tn1546 from enterococcus faecium BM4147 confers resistance to teicoplanin. Gene, 154(1), 87–92. http://doi.org/10.1016/0378-1119(94)00851-I

[72] Hava, D. L., & Camilli, A. (2002). Large-scale identification of serotype 4 Streptococcus pneumoniae virulence factors. Molecular Microbiology, 45, 1389–1406. http://doi.org/3106 [pii]

[73] Humphries, H. E., & High, N. J. (2002). The role of licA phase variation in the pathogenesis of invasive disease by Haemophilus influenzae type b. FEMS Immunology and Medical Microbiology, 34(3), 221–230. http://doi.org/10.1016/S0928-8244(02)00394-2

[74] Serino, L., & Virji, M. (2000). Phosphorylcholine decoration of lipopolysaccharide differentiates commensal Neisseriae from pathogenic strains: Identification of licA-type genes in

commensal Neisseriae. Molecular Microbiology, 35(6), 1550–1559. http://doi.org/10.1046/j.1365-2958.2000.01825.x
